# The role of diet in the development of breast cancer: a case-control study of patients with breast cancer, benign epithelial hyperplasia and fibrocystic disease of the breast.

**DOI:** 10.1038/bjc.1991.268

**Published:** 1991-07

**Authors:** D. M. Ingram, E. Nottage, T. Roberts

**Affiliations:** Queen Elizabeth II Medical Centre, Perth, Western Australia.

## Abstract

A case-control study was undertaken to investigate the role of diet in women with breast cancer, and in two groups of women with benign breast disease: epithelial hyperplasia, and fibrocystic disease without hyperplasia. The study provides data which suggest that the consumption of red meat, savoury meals (pizza, pies, stew, etc.) and of starches is disadvantageous, while the consumption of chicken and fish, and of fruit, appears to be beneficial. These patterns were present for both the breast cancer patients and the patients with benign epithelial hyperplasia. One-third of breast cancer patients had changed their diet after their diagnosis, compared to only around 12% in controls and patients with benign breast disease. Overall, the women studied had changed their diet to reduce their intake of sugars, dairy products and meat, and increased their intake of poultry, fish, fruit and vegetables over the past decade; but the breast cancer group was less likely to have made this change.


*Br. J. Cancer* (1991), **64**, 187–191 © Macmillan Press Ltd., 1991

### The role of diet in the development of breast cancer: a case-control study of patients with breast cancer, benign epithelial hyperplasia and fibrocystic disease of the breast

D.M. Ingram, E. Nottage & T. Roberts

*Queen Elizabeth II Medical Centre, Perth, Western Australia 6009.*

**Summary** A case-control study was undertaken to investigate the role of diet in women with breast cancer, and in two groups of women with benign breast disease: epithelial hyperplasia, and fibrocystic disease without hyperplasia.

The study provides data which suggest that the consumption of red meat, savoury meals (pizza, pies, stew, etc.) and of starches is disadvantageous, while the consumption of chicken and fish, and of fruit, appears to be beneficial. These patterns were present for both the breast cancer patients and the patients with benign epithelial hyperplasia.

One-third of breast cancer patients had changed their diet after their diagnosis, compared to only around 12% in controls and patients with benign breast disease. Overall, the women studied had changed their diet to reduce their intake of sugars, dairy products and meat, and increased their intake of poultry, fish, fruit and vegetables over the past decade; but the breast cancer group was less likely to have made this change.

Before we can devise ways of preventing breast cancer from developing, causative factors must be identified. Of those identified to date, diet has aroused the greatest interest and debate because of its potential for preventing breast cancer.

The early international correlation studies clearly associated animal fat consumption with breast cancer mortality (Armstrong & Doll, 1975), yet case-control studies have, on the whole, failed to confirm that a diet rich in fat increases the risk of developing breast cancer (Goodwin & Boyd, 1987). There are a number of reasons why case-control studies may not detect differences in diet between breast cancer patients and their controls, if such differences do in fact exist. Amongst them is the likelihood that any influence of nutrition on the breast to increase its susceptibility to cancer probably takes place a decade or more before the breast cancer becomes clinically apparent (Ingram, 1981), and in the intervening period the patient's dietary habits may have changed.

In an attempt to overcome this problem, we have undertaken a case-control study which includes not only breast cancer patients and their controls, but also a group at increased risk of developing breast cancer, women with benign epithelial hyperplasia of the breast (BEH). Hyperplasia of the breast epithelium has been identified as a change which probably precedes breast malignancy (Dupont & Page, 1985), and although the time interval is uncertain, it is likely to be at least a decade. In addition, patients at low risk of developing breast cancer, women with benign fibrocystic disease of the breast without histological evidence of hyperplasia (FCD), were studied as a control group, as well as a group of community controls. If nutrition were to play a part in breast cancer development, one would expect the dietary intake of the low-risk group (FCD) to differ little from controls, while that of the higher-risk group (BEH) would show differences which might give an insight into dietary habits long before breast cancer had developed, and into how diet may influence the very early stages of breast cancer development.

Correspondence: D.M. Ingram, University Department of Surgery, Queen Elizabeth II Medical Centre, Nedlands, Western Australia 6009.

Received 29 August 1990; and in revised form 13 February 1991.

### Method

### *Study population*

Five hundred and fourteen women were studied in the period February 1985 to August 1987. Cases were identified from the pathology records at the Queen Elizabeth II Medical Centre, Perth, Western Australia, and were contacted after first gaining the approval of their surgeons. The histopathology of each case was reviewed by a single pathologist (A.R.), who categorised the cases into invasive breast cancer (IBC), BEH or FCD according to set criteria. Patients with other pathologies were not included. Controls were identified from the electoral roll, and were contacted in the same manner as cases, by way of a letter requesting their participation in a health survey, but without specific mention of breast disease. If the letter was not answered, it was followed by a telephone call. None of the patients and only 5.5% of controls could not be contacted. All breast cancer patients, 78% of benign breast disease patients, and 78% of contactable controls, agreed to participate in the study.

Of the 514 women who initially agreed to participate, 99 breast cancer patients, 91 patients with benign epithelial hyperplasia, 95 patients with fibrocystic disease and 209 controls were included in the study. Twenty subjects were excluded from the study: two died before data could be collected, one was pregnant, ten withdrew for personal reasons, and in seven instances the dietary questionnaire was so incomplete as to be not usable, even after further attempts to complete it.

Cases were matched to controls by age (5-year age group) and area of residence (electoral district). Where possible, more than one control was used for each case. In addition, the patients with fibrocystic disease were matched to those with benign epithelial hyperplasia, so these groups shared the same controls.

### *Interview*

A structured intervew was conducted in the subject's home by a single interviewer (E.N.), 3 months after their operation. Although the interviewer was blind to the diagnosis, in the course of the interview it would often become apparent which were the breast cancer patients.

Data were collected as regards age, menstrual status, ethnic origin, occupation, risk factors for breast cancer, previous breast disease, other major illnesses and medications. Each woman was asked whether her diet had changed for any reason in the past 3 months. In addition, each was asked how her eating patterns had changed over the past 10 years, asking specifically regarding consumption of red meats, poultry and fish, saturated fats, polyunsaturated fats, fruit and vegetables, dairy products, cereal products, sugars and prepared or convenience foods. Height and weight were measured using the same set of scales.

After explanation and demonstration of standard portion sizes, each subject was requested to complete a food frequency questionnaire, based on current consumption, in their own time and return it by mail. Any problems were resolved per telephone. The food frequency questionnaire has been tested previously, and came with a commerical analysis package (FREQUAN, the Division of Human Nutrition, Commonwealth Scientific and Industrial Research Organisation, South Australia) (Baghurst & Record, 1984). This program identifies 179 different foods, and analysis is based on both portion size and frequency of consumption. The program generates a breakdown of consumption of nutrients as well as the consumption of the main food groups. In addition, the proportion of total energy derived from each of the major nutrients was calculated.

*Statistical analysis*

Quartiles for each nutrient and food group were derived from the control populations, and the odds ratio and 95% confidence limits determined by conditional logistic regression. These were determined both for quartiles and at the median level of consumption. Significant associations were recalculated after adjusting for known risk factors for breast cancer: parity, first-degree family history of breast cancer, age at menarche and body mass index.

The mean consumption of each nutrient and food group were calculated for each patient group and their control. Means were compared by one-way analysis of variance.

### Results

*Study population (Table I)*

A comparison of patient and control characteristics and risk factors for each of the study groups is shown in Table I. The only significant differences between cases and controls were for family history and indices of obesity. Nineteen per cent of cancer patients had a first-degree relative with breast cancer, compared with 8.7% of the controls ($P<0.05$). Ten per cent of BEH patients had a positive family history of breast cancer, compared with 6% of controls, but this did not reach significance. BEH patients weighed less and had a lower body mass index than controls. These results have been presented and discussed previously (Ingram *et al.*, 1989). The breast cancer patients' lower parity when compared with controls, and their lower age at menarche, did not reach statistical significance.

*Estimations of risk (Table II)*

After estimation of the odds ratio for consumption of each nutrient, the only significant finding was for the proportion of energy derived from sugar, this being protective for benign epithelial hyperplasia. When examined by quartiles, an additional significant finding was of a protective effect for vitamin C consumption, with fibrocystic disease in the highest quartile of consumption (O.R 0.4, c.l. 0.1–0.9). An increase in risk was demonstrated, however, for both the cancer and benign epithelial groups for starch consumption (cancer O.R. 2.0, c.l. 0.9–4.4; BEH O.R. 2.1, c.l. 0.9–4.7), and for monounsaturated fat consumption (cancer O.R. 1.9, c.l. 0.8–4.2; BEH O.R. 2.3, c.l. 0.9–6.3) for the highest quartiles of consumption. These did not quite reach significance.

Analysis of consumption of food groups demonstrated that, for the cancer patients, there was a significant increase in risk with consumption of red meats and savoury snacks; for the BEH patients, a protective effect for consumption of eggs, chicken and seafood; while the FCD group also demonstrated the protective effect of egg consumption. When considering the highest quartile of food consumption, additional significant findings were of a reduced risk in the BEH group with consumption of fruit (O.R. 0.4, c.l. 0.2–0.9) and leafy and orange-red vegetables (O.R. 0.4, c.l. 0.2–0.9), and also for the consumption of yellow-orange fruit in the FCD group (O.R. 0.4, c.l. 0.1–0.9).

The significant associations were recalculated after adjusting for possible confounding variables. The previously significant elevated odds ratios for red meat and for snacks (Table IIc) fell to just below significant levels. Other significant odds ratios were not affected.

*Comparison of mean consumption between cases and controls (Table III)*

Cancer patients had significantly greater consumption of retinol, red meats and savoury foods compared to controls. The BEH patients similarly consumed more savoury foods, but this did not reach significance.

Patients with benign epithelial hyperplasia consumed significantly more starches, both when calculated as actual consumption and when calculated as a proportion of total energy consumption. The cancer patients similarly consumed more starches, but again not quite reaching statistical significance. The increase in starch consumption appeared to come from the increased consumption of starchy vegetables.

Benign epithelial hyperplasia patients consumed significantly less chicken and seafood and less fruit than controls. Again, similar patterns of consumption are seen for the cancer patients, but also not quite to a significant level.

The fibrocystic group were found to consume significantly less vitamin C and yellow-orange fruit than controls, and more polyunsaturated fats.

*Change in diet (Tables III and IV)*

As there was concern that a recent diagnosis of breast cancer might have influenced a subject's diet, all subjects were asked

**Table I** Comparison of breast cancer risk factors for cases and controls

| | | | *Group* | | |
|---|---|---|---|---|---|
| | *Breast cancer* | *Cancer control* | *Benign epithelial hyperplasia* | *Fibrocystic disease* | *Benign control* |
| Age (years) (mean ± s.e.m.) | 55.9 ± 1.3 | 55.5 ± 1.3 | 43.5 ± 1.1 | 43.3 ± 1.0 | 43.7 ± 1.0 |
| Age range (years) | 25–86 | 22–86 | 19–72 | 19–71 | 20–72 |
| Parity (number children) | 2.50 ± 0.1 | 2.55 ± 0.1 | 2.41 ± 0.1 | 2.30 ± 0.1 | 2.43 ± 0.1 |
| First-degree family history (%) | 19.0 ± 3.9[a] | 8.7 ± 2.4 | 10.0 ± 3.1 | 7.3 ± 2.6 | 6.0 ± 2.1 |
| Weight (kg) | 64.9 ± 1.1 | 64.2 ± 1.2 | 62.1 ± 1.0[a] | 63.8 ± 1.2 | 65.6 ± 1.4 |
| Body mass index (kg $m^{-2}$) | 25.82 ± 0.5 | 25.46 ± 0.4 | 23.45 ± 0.4[b] | 24.09 ± 0.4 | 25.2 ± 0.4 |
| Age at menarche (years) | 13.02 ± 0.1 | 13.42 ± 0.1 | 13.10 ± 0.1 | 13.12 ± 0.1 | 13.23 ± 0.1 |
| Age at menopause (years) | 48.24 ± 0.7 | 48.73 ± 0.7 | 48.88 ± 1.6 | 50.4 ± 1.4 | 46.3 ± 2.1 |

[a]$P<0.05$; [b]$P<0.01$.

**Table II** Estimation of odds ratio and 95% confidence limits for consumption of nutrients and food groups for each of the patient groups studied

| Nutrient | Breast cancer O.R. | Breast cancer 95% CI | Breast cancer excluding diet change subjects O.R. | Breast cancer excluding diet change subjects 95% CI | Benign epithelial hyperplasia O.R. | Benign epithelial hyperplasia 95% CI | Fibrocystic disease O.R. | Fibrocystic disease 95% CI |
|---|---|---|---|---|---|---|---|---|
| *A. Actual consumption* | | | | | | | | |
| Total energy | 1.1 | 0.6–2.0 | 1.2 | 0.6–2.4 | 0.8 | 0.5–1.5 | 0.9 | 0.5–1.5 |
| Total carbohydrates | 1.5 | 0.8–2.7 | 1.4 | 0.7–2.8 | 0.9 | 0.5–1.6 | 1.1 | 0.7–1.9 |
| sugar | 1.1 | 0.6–1.9 | 0.9 | 0.4–1.7 | 0.7 | 0.4–1.2 | 1.0 | 0.6–1.6 |
| starches | 1.5 | 0.9–2.6 | 2.0 | 1.0–3.8[a] | 1.3 | 0.7–2.3 | 1.1 | 0.6–2.0 |
| Total protein | 1.4 | 0.8–2.4 | 1.4 | 0.7–2.9 | 0.9 | 0.5–1.6 | 0.8 | 0.5–1.6 |
| Total fats | 1.4 | 0.8–2.5 | 1.7 | 0.8–3.4 | 1.1 | 0.6–2.0 | 0.8 | 0.5–1.5 |
| saturated | 1.0 | 0.6–1.8 | 1.6 | 0.8–3.1 | 0.9 | 0.5–1.7 | 0.9 | 0.5–1.5 |
| monounsaturated | 1.6 | 0.9–2.9 | 2.3 | 1.1–4.7[a] | 1.1 | 0.6–2.0 | 0.9 | 0.5–1.5 |
| polyunsaturated | 0.9 | 0.4–1.7 | 1.0 | 0.4–2.2 | 1.1 | 0.6–2.0 | 0.8 | 0.5–1.4 |
| Fibre | 1.5 | 0.9–2.6 | 1.1 | 0.6–2.1 | 0.8 | 0.5–1.5 | 0.7 | 0.4–1.3 |
| Vitamins | | | | | | | | |
| Retinol | 1.0 | 0.6–1.7 | 1.1 | 0.6–2.1 | 1.2 | 0.6–2.1 | 1.3 | 0.7–2.3 |
| Betacarotene | 0.8 | 0.5–1.4 | 0.8 | 0.4–1.7 | 0.9 | 0.5–1.7 | 0.7 | 0.4–1.2 |
| B1 | 1.2 | 0.7–2.2 | 1.2 | 0.6–2.3 | 0.7 | 0.4–1.3 | 0.6 | 0.4–1.1 |
| B6 | 1.3 | 0.7–2.3 | 1.2 | 0.6–2.5 | 1.0 | 0.5–1.7 | 0.6 | 0.4–1.1 |
| C | 1.5 | 0.8–2.6 | 1.2 | 0.6–2.4 | 0.8 | 0.4–1.4 | 0.6 | 0.4–1.2 |
| *B. Consumption as a proportion of total energy* | | | | | | | | |
| Sugars | 1.0 | 0.6–1.8 | 1.1 | 0.7–1.9 | 0.5 | 0.3–0.9[a] | 0.7 | 0.4–1.2 |
| Starches | 1.4 | 0.8–2.4 | 1.3 | 0.8–2.3 | 1.3 | 0.7–2.3 | 1.1 | 0.6–1.9 |
| Protein | 0.6 | 0.4–1.1 | 0.7 | 0.4–1.2 | 1.0 | 0.6–1.8 | 1.0 | 0.6–1.8 |
| Total fat | 1.3 | 0.7–2.4 | 1.2 | 0.7–2.2 | 1.1 | 0.6–2.0 | 1.3 | 0.7–2.4 |
| saturated | 1.0 | 0.6–1.9 | 1.0 | 0.6–1.8 | 0.8 | 0.5–1.5 | 0.8 | 0.4–1.4 |
| monounsaturated | 1.4 | 0.7–2.6 | 1.3 | 0.7–2.4 | 1.3 | 0.6–2.8 | 1.2 | 0.7–2.1 |
| polyunsaturated | 0.6 | 0.3–1.1 | 0.6 | 0.3–1.1 | 1.5 | 0.8–2.9 | 1.1 | 0.6–1.9 |
| *C. Food group* | | | | | | | | |
| Cereal products | 0.9 | 0.5–1.6 | 0.8 | 0.4–1.7 | 1.5 | 0.9–2.7 | 1.1 | 0.4–1.4 |
| Cakes, desserts, sweets, jam, etc | 0.7 | 0.5–1.5 | 1.1 | 0.6–2.1 | 0.9 | 0.5–1.7 | 0.8 | 0.4–1.4 |
| Dairy products (total) | 1.3 | 0.7–2.2 | 0.9 | 0.4–1.8 | 0.7 | 0.4–1.2 | 1.1 | 0.6–1.9 |
| Milk and milk products | 0.9 | 0.5–1.6 | 0.6 | 0.3–1.2 | 0.6 | 0.3–1.1 | 0.9 | 0.5–1.6 |
| Eggs | 1.0 | 0.6–1.8 | 1.3 | 0.7–2.5 | 0.4 | 0.2–0.8[b] | 0.5 | 0.3–0.9[a] |
| Butter and margarine | 1.2 | 0.7–2.0 | 2.3 | 1.1–5.1[a] | 1.2 | 0.7–2.2 | 0.8 | 0.5–1.3 |
| Meat (total) | 1.6 | 0.9–2.8 | 2.0 | 1.0–4.0[a] | 0.8 | 0.4–1.3 | 0.8 | 0.4–1.4 |
| Red meats | 1.8 | 1.0–3.2[a] | 1.9 | 0.9–3.9 | 0.9 | 0.5–1.5 | 0.7 | 0.4–1.2 |
| Chicken and seafood | 0.9 | 0.5–1.6 | 0.9 | 0.4–1.8 | 0.5 | 0.3–0.9[a] | 0.6 | 0.4–1.1 |
| Savoury foods (total) | 1.7 | 1.0–2.9 | 1.6 | 0.8–3.3 | 1.5 | 0.8–2.7 | 0.8 | 0.4–1.4 |
| Meals, e.g. pizza, stew, pies, etc. | 0.9 | 0.6–1.8 | 1.0 | 0.5–1.9 | 1.0 | 0.6–1.8 | 1.0 | 0.6–1.8 |
| Snacks, e.g. crisps, nuts, biscuits | 2.0 | 1.0–3.8[a] | 0.9 | 0.4–2.1 | 0.9 | 0.5–1.6 | 0.8 | 0.5–1.6 |
| Fruit (total) | 0.9 | 0.5–1.6 | 0.5 | 0.2–1.2 | 0.8 | 0.4–1.5 | 1.1 | 0.6–2.0 |
| Yellow and orange fruit | 1.1 | 0.6–2.0 | 1.2 | 0.6–2.6 | 0.7 | 0.4–1.3 | 0.7 | 0.4–1.3 |
| Other fruit | 1.0 | 0.5–1.8 | 0.5 | 0.2–1.2 | 0.6 | 0.3–1.1 | 0.9 | 0.5–1.7 |
| Vegetables (total) | 1.4 | 0.8–2.4 | 1.6 | 0.8–3.3 | 1.2 | 0.7–2.1 | 0.8 | 0.4–1.3 |
| Leafy and orange/red vegetables | 1.0 | 0.6–1.7 | 1.1 | 0.6–2.2 | 0.7 | 0.4–1.2 | 0.6 | 0.4–1.1 |
| Starchy vegetables | 0.9 | 0.5–1.6 | 1.1 | 0.5–2.3 | 1.7 | 1.0–3.2 | 1.2 | 0.7–2.1 |

Estimates are based on the median consumption of each nutrient by control subjects. [a]$P<0.05$; [b]$P<0.01$.

whether their diet had changed for any reason over the past 3 months. Around one-third of cancer patients admitted to recent dietary change, while only little more than 10% of patients with benign biopsies and controls admitted to change in diet (Table IV).

As there was a considerable difference between the number of cancer patients and their controls who had changed their diet recently, the statistics were recalculated for these groups after exclusion of those who had changed their diet (Table III). This reanalysis resulted in consumption of starches (O.R. 2.0) and of monounsaturated fats (O.R. 2.3) from the nutrient analysis, and of butter and margarine (O.R. 2.3) and mean (O.R. 2.0) from the food group analysis, now becoming significant variables (Table II). Reanalysis of the mean consumption (Table III) had little effect on the overall results, but did increase the mean consumption of fats and reduce the mean consumption of fibre and vitamins for the cancer patients. These changes were also seen in the food group analyses where the mean consumption of cakes etc., butter and margarine, meat and savoury meals increased and the consumption of fruit decreased.

In addition, each woman was asked how her eating patterns had changed over the past 10 years (Table V). In general, the trend was towards eating less red meat, saturated fat, dairy products and sugars, and more poultry and fish, unsaturated fats and fruit and vegetables. Although there was little difference between the cancer patients and other groups in these trends, for every food group the cancer patients were more likely to have scored 'no change' in their diet over the past 10 years.

### Discussion

The results of studies to date investigating associations between diet and breast cancer are inconsistent. The original concept that diet, and particularly dietary fat, might be related to breast cancer development came from international correlation studies. All have demonstrated significant positive correlations between fat intake and breast cancer mortality. A strong correlation, however, does not mean that the association is causal. Fat intake is, in general, an indicator of affluence, and there are many other differences apparent between countries with a high and low breast cancer mortality. National or regional studies have, for the most part, also shown a significant positive correlation between fat intake and breast cancer mortality.

With case-control studies, very few have found significant associations between dietary fat and breast cancer risk, although a number of studies identified consumption of fat-containing foods such as meat, butter and margarine, and breast cancer risk. Goodman and Boyd (1987) published a

**Table III** Mean (±s.e.m.) consumption of nutrients and food groups for each study group and their controls

| *Nutrient* | *Unit* | *Breast cancer* | *Breast cancer excluding diet change subjects* | *Cancer control* | *Benign epithelial hyperplasia* | *Fibrocystic disease* | *Benign control* |
|---|---|---|---|---|---|---|---|
| A. *Actual consumption* | | | | | | | |
| Total energy | kJ | 8316±271 | 8411±322 | 8022±217 | 8592±324 | 8142±268 | 8218±224 |
| Total carbohydrate | g day$^{-1}$ | 225±8 | 221±10 | 218±7 | 238±12 | 223±8 | 228±7 |
| sugars | g day$^{-1}$ | 124±6 | 121±7 | 126±5 | 135±10 | 129±6 | 135±6 |
| starches | g day$^{-1}$ | 100±4.0 | 100±5 | 91±3.1 | 103±4[a] | 93±4 | 92±3 |
| Total protein | g day$^{-1}$ | 84±3 | 85±3 | 83±2 | 85±3 | 83±2 | 85±2 |
| Total fats | g day$^{-1}$ | 72.1±2.8 | 75.2±3.3 | 69.0±2.3 | 75.7±3.2 | 70.7±2.9 | 70.6±2.5 |
| saturated | g day$^{-1}$ | 31.4±1.5 | 33.4±1.8 | 29.7±1.2 | 32.6±1.7 | 29.3±1.3 | 30.6±1.3 |
| monounsaturated | g day$^{-1}$ | 27.2±1.1 | 28.3±1.2 | 25.6±0.9 | 28.3±1.2 | 26.2±1.1 | 26.4±0.9 |
| polyunsaturated | g day$^{-1}$ | 13.5±0.7 | 13.4±0.7 | 13.7±0.6 | 14.8±0.7 | 15.2±0.9 | 13.6±0.5 |
| Fibre | g day$^{-1}$ | 26.9±1.1 | 24.5±1.0 | 25.6±1.0 | 25.4±1.1 | 24.2±0.9 | 26.6±1.0 |
| Vitamins | | | | | | | |
| Retinol | unit day$^{-1}$ | 931±120[a] | 849±127 | 652±75 | 662±83 | 792±95 | 683±86 |
| Betacarotene | μg day$^{-1}$ | 7772±464 | 7370±482 | 7356±351 | 7403±497 | 7232±395 | 8076±433 |
| B1 | mg day$^{-1}$ | 1.41±0.05 | 1.33±0.05 | 1.31±0.04 | 1.34±0.05 | 1.29±0.04 | 1.37±0.04 |
| B6 | mg day$^{-1}$ | 1.81±0.06 | 1.74±0.06 | 1.72±0.05 | 1.71±0.06 | 1.71±0.05 | 1.79±0.05 |
| C | mg day$^{-1}$ | 174±8.7 | 162±9 | 162±8.6 | 159±9 | 149±6[b] | 184±10 |
| B. *Consumption as a proportion of total energy* | | | | | | | |
| Sugars | % | 24.0±0.7 | 23.0±0.9 | 25.1±0.7 | 23.8±0.9 | 25.6±0.8 | 26.4±0.8 |
| Starches | % | 19.3±0.4 | 19.0±0.5 | 18.3±0.4 | 19.8±0.6[b] | 18.3±0.4 | 17.9±0.4 |
| Protein | % | 17.6±0.3 | 17.4±0.4 | 17.9±0.3 | 17.3±0.3 | 17.7±0.3 | 18.0±0.3 |
| Fats | % | 31.6±0.6 | 32.8±0.6 | 31.6±0.5 | 32.4±0.6 | 31.6±0.6 | 31.3±0.5 |
| saturated | % | 13.6±0.4 | 14.4±0.4 | 13.5±0.3 | 13.7±0.4 | 13.1±0.3 | 13.4±0.3 |
| monounsaturated | % | 12.0±0.2 | 12.4±0.3 | 11.8±0.2 | 12.1±0.2 | 11.7±0.2 | 11.7±0.2 |
| polyunsaturated | % | 6.09±0.25 | 6.0±0.3 | 6.33±0.21 | 6.62±0.25 | 6.78±0.27[a] | 6.16±0.19 |
| C. *Food group* | | | | | | | |
| Cereal products | g day$^{-1}$ | 71.6±3.6 | 68.9±4.2 | 68.4±2.8 | 73.5±3.5 | 67.8±3.7 | 66.1±2.9 |
| Cakes, desserts, sweets, jam, etc | g day$^{-1}$ | 46.5±4.6 | 52.2±6.2 | 54.8±5.4 | 54.7±8.4 | 41.4±4.0 | 49.5±5.3 |
| Dairy products (total) | g day$^{-1}$ | 65.2±3.6 | 64.8±4.5 | 65.6±2.9 | 70.1±4.9 | 68.9±3.0 | 67.6±2.9 |
| Milk and milk products | g day$^{-1}$ | 47.4±3.2 | 46.0±4.0 | 48.9±2.6 | 52.9±4.5 | 53.2±2.9 | 51.1±2.6 |
| Eggs | g day$^{-1}$ | 4.9±0.5 | 5.5±0.6 | 4.5±0.4 | 4.4±0.6 | 4.1±0.4 | 4.8±0.4 |
| Butter and margarine | g day$^{-1}$ | 12.9±0.8 | 13.4±0.9 | 12.3±0.7 | 12.8±0.9 | 11.6±1.0 | 11.6±0.7 |
| Meat (total) | g day$^{-1}$ | 56.5±2.7 | 58.0±1.8 | 52.6±2.0 | 53.4±2.5 | 54.1±2.4 | 55.1±2.0 |
| Red meats | g day$^{-1}$ | 41.4±2.6[a] | 43.5±2.8[a] | 35.3±1.7 | 39.7±2.5 | 39.0±2.3 | 38.4±1.8 |
| Chicken and seafood | g day$^{-1}$ | 15.1±0.8 | 15.6±1.1 | 17.3±1.0 | 13.7±1.0[a] | 15.1±1.1 | 16.6±1.0 |
| Savoury foods (total) | g day$^{-1}$ | 48.2±3.1[a] | 47.8±3.7[a] | 39.8±2.3 | 49.6±3.2 | 42.4±2.6 | 44.4±2.5 |
| Meals, e.g. pizza, stew, pies, etc | g day$^{-1}$ | 34.9±2.8 | 36.3±3.1[a] | 29.7±1.8 | 39.4±3.0 | 32.8±2.0 | 33.1±1.9 |
| Snacks, e.g. crisps, nuts, biscuits | g day$^{-1}$ | 13.3±1.8 | 11.4±1.8 | 10.1±1.1 | 10.2±1.3 | 10.6±1.3 | 11.4±1.2 |
| Fruit (total) | g day$^{-1}$ | 51.7±3.5 | 45.1±4.6 | 54.1±3.7 | 44.9±3.7[a] | 48.7±3.4 | 58.2±4.7 |
| Yellow and orange fruit | g day$^{-1}$ | 19.8±2.0 | 22.1±1.8 | 19.3±1.7 | 17.3±1.7 | 14.8±1.2[a] | 20.8±1.9 |
| Other fruit | g day$^{-1}$ | 31.9±2.3 | 26.7±2.4 | 34.8±2.5 | 27.5±2.4[a] | 33.9±2.9 | 37.3±3.7 |
| Vegetables (total) | g day$^{-1}$ | 55.1±3.1 | 55.3±3.7 | 49.5±2.1 | 55.6±2.8 | 52.7±3.0 | 52.4±1.8 |
| Leafy and orange-red vegetables | g day$^{-1}$ | 22.0±1.5 | 22.1±1.8 | 19.8±1.1 | 19.1±1.4 | 20.0±1.3 | 21.9±1.1 |
| Starchy vegetables | g day$^{-1}$ | 33.1±2.3 | 33.2±2.7 | 29.7±1.5 | 36.5±2.5[a] | 32.7±2.1 | 30.5±1.4 |

[a]$P<0.05$; [b]$P<0.01$.

**Table IV** Proportion of study subjects who stated they had changed their diet in the past 3 months

| | *Breast cancer* | *Cancer controls* | *Benign epithelial hyperplasia* | *Fibrocystic disease* | *Benign controls* |
|---|---|---|---|---|---|
| Changed diet | 32% | 11% | 13% | 12% | 11% |
| No change in diet | 68% | 89% | 87% | 86% | 89% |

**Table V** Change in diet over the past decade

| | *Now eat less* | | *No change* | | *Now eat more* | |
|---|---|---|---|---|---|---|
| | *Total group* | *Cancer patients* | *Total group* | *Cancer patients* | *Total group* | *Cancer patients* |
| Cereal products | 20% | 16% | 64% | 71% | 16% | 13% |
| Sugars | 47% | 34% | 52% | 63% | 1% | 3% |
| Dairy products | 31% | 23% | 64% | 71% | 5% | 6% |
| Saturated fats | 47% | 40% | 51% | 60% | 2% | 0% |
| Polyunsaturated fats | 14% | 5% | 56% | 67% | 30% | 28% |
| Red meat | 59% | 50% | 37% | 45% | 4% | 5% |
| Poultry/fish | 6% | 9% | 45% | 50% | 49% | 41% |
| Prepared/convenience food | 15% | 14% | 70% | 78% | 15% | 18% |
| Fruit and vegetables | 3% | 2% | 59% | 70% | 38% | 28% |

The breast cancer patients were around 10% less likely to have made a change in their diet.

detailed critical analysis of all studies published on the subject to that time. Howe *et al.* (1990) have recently conducted a combined analysis of the original data from 12 case-control studies. Their results show a consistent, statistically significant, positive association between breast cancer risk and saturated fat consumption in postmenopausal women, the relative risk for highest *vs* lowest quintiles being 1.46 ($P<0.0001$). In addition, a consistent protective effect for a number of markers of fruit and vegetable intake was demonstrated. For Vitamin C, the relative risk of highest *vs* lowest quintile was 0.69 ($P<0.0001$).

At odds with these results are those of the two large North American cohort studies. A study of 89,538 nurses by Willett *et al.* (1987) and of 5,485 women by Jones *et al.* (1987) failed to find any evidence of a positive association between breast cancer risk and fat intake. Indeed, the relative risk for the highest *vs* lowest quintile of saturated fat in postmenopausal women was only 0.79 in Willett's study, and similarly Jones' study showed an apparent protective effect of high fat intake. To resolve the discrepancy in results between these cohort studies and the case-control studies and correlation studies, further analytic epidemiological data need to be acquired.

A number of findings of interest have arisen from the study. The consumption of starches, particularly from vegetables, appeared to be disadvantageous, and is seen in both the BEH and cancer groups. Such a finding is difficult to explain, and no mechanisms is apparent. The consumption of savoury foods such as pizzas, stews, etc and the consumption of red meat appeared to be detrimental, while the consumption of chicken and seafood were beneficial. In general, these findings are also present in both the BEH and cancer groups. Unfortunately, the dietary analysis program does not allow separation of the poultry and fish groups so that the apparent beneficial effect of these could be explored further. The above findings would suggest that the type of fat consumed may be important; however, in the analysis by breakdown into nutrients, it is only the consumption of monounsaturated fats which differs between cases and controls, and then only at the highest quartile of consumption.

The consumption of fruit appears to be beneficial, again these findings being apparent for both the BEH and the breast cancer groups. Presumably this beneficial effect is through anti-oxidants in the food, but surprisingly no significant differences for beta-carotene or vitamin C are seen between cases and controls, although the BEH group does have a considerably lower consumption of these than the controls. As hypothesised, only occasional significant associations were seen for the FCD group, and in general consumption of nutrients and food groups were similar to the controls.

If recommendations were to be made from these data as to a diet which might help reduce the incidence of breast cancer, they would be similar to those of the generally promoted 'healthy' diet of a reduced consumption of red meats and prepared foods, and an increased consumption of poultry, fish, fruit and vegetables. It is of note that, while the data in Table V suggests that the populace is already moving towards such a diet, the women in the breast cancer group were the least likely to have made changes over this time.

We would like to thank Dr Bruce Armstrong, for his contribution to the concept and design of the study; Mrs Peta Diffen, for assistance with data collation; Mrs Alison Ginsberg, for typing the manuscript; the Western Australian surgeons who allowed their patients to be interviewed; and finally, the women who unselfishly gave of their time to take part in the study.

### References

ARMSTRONG, B. & DOLL, R. (1975). Environmental factors and breast cancer incidence and mortality in different countries, with special reference to dietary practices. *Int. J. Cancer*, **15**, 617.

BAGHURST, R.I. & RECORD, S.J. (1984). A computerised dietary analysis system for use with diet diaries or food frequency questionnaires. *Comm. Health Stud.*, **8**, 11.

DUPONT, W.D. & PAGE, D.L. (1985). Risk factors for breast cancer in women with proliferative breast disease. *N. Engl. J. Med.*, **312**, 146.

GOODWIN, P.J. & BOYD, N.F. (1987). Critical appraisal of the evidence that dietary fat intake is related to breast cancer risk in humans. *JNCI*, **79**, 473

HOWE, G.R., HIROHATA, I., HISLOP, I.G. & 9 others (1990). Dietary factors and risk of breast cancer: combined analysis of 12 case-control studies. *JNCI*, **82**, 561.

INGRAM, D.M. (1981). Trends in diet and breast cancer mortality in England and Wales 1928–1977. *Nutr. Cancer*, **3**, 75.

INGRAM, D., NOTTAGE, E., NG, S., SPARROW, L., ROBERTS, A. & WILLCOX, D. (1989). Obesity and breast disease: the role of the female sex hormones. *Cancer*, **64**, 1049.

JONES, D.Y., SCHATZKIN, A., GREEN, S.B. & 5 others (1987). Dietary fat and breast cancer in the National Health and Nutrition Examination Survey 1 epidemiological follow-up study. *JNCI*, **79**, 465.

WILLETT, W.C., STAMPFER, M.J., COLDITZ, G.A., ROSNER, B.A., HENNEKENS, C.H. & SPEIZER, F.E. (1987). Dietary fat and the risk of breast cancer. *N. Engl. J. Med.*, **316**, 22.


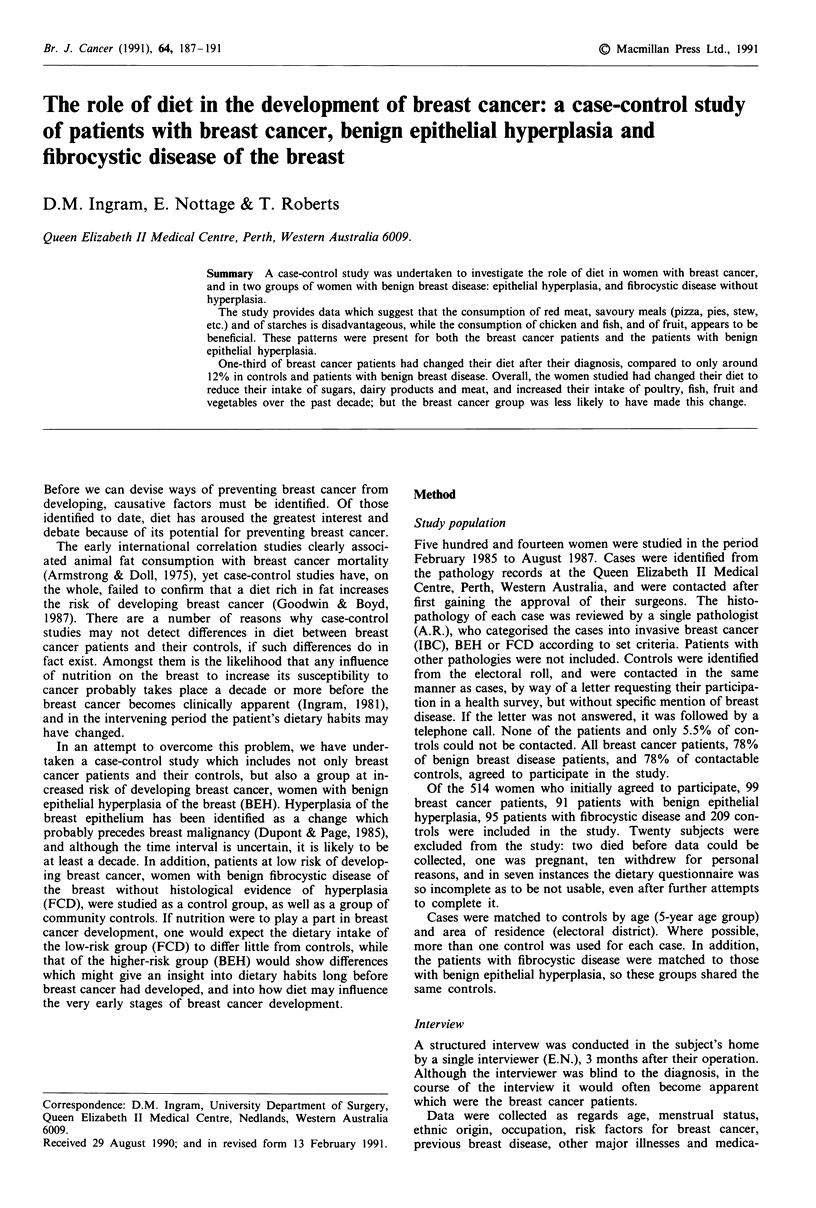

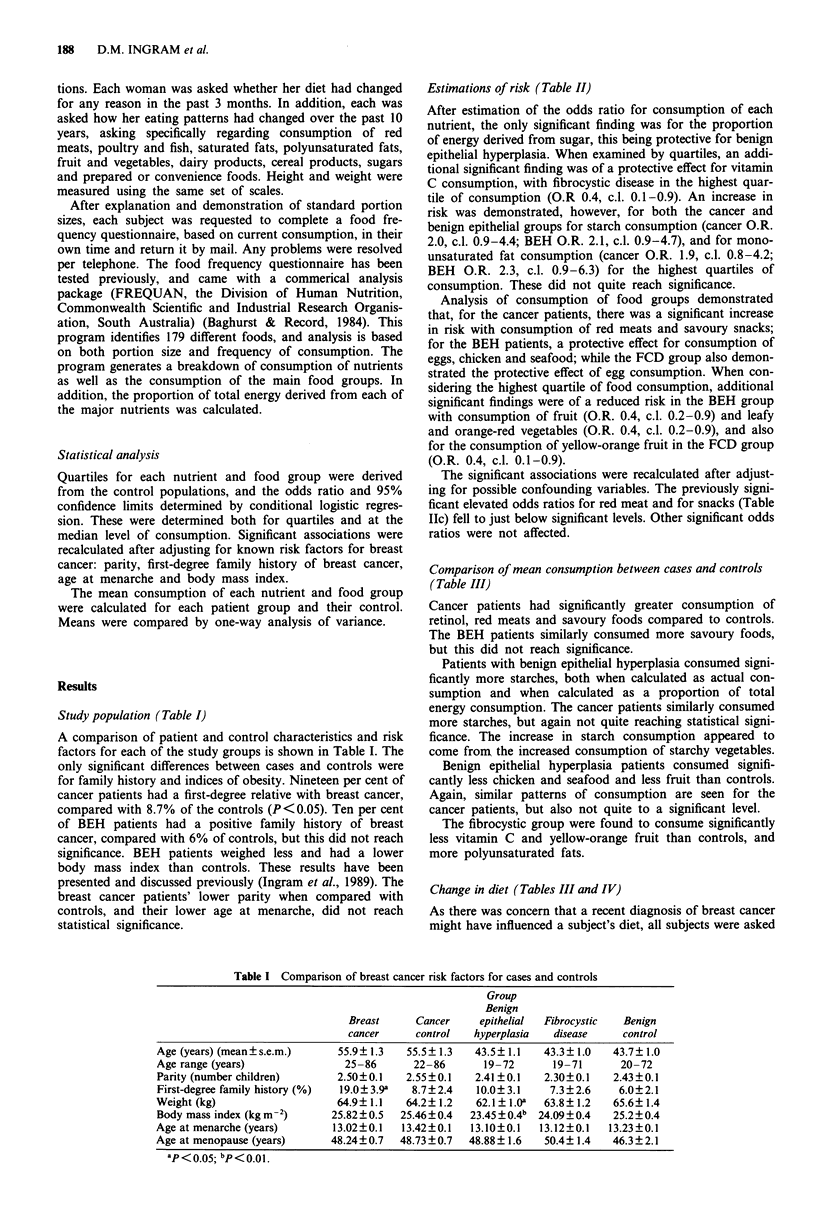

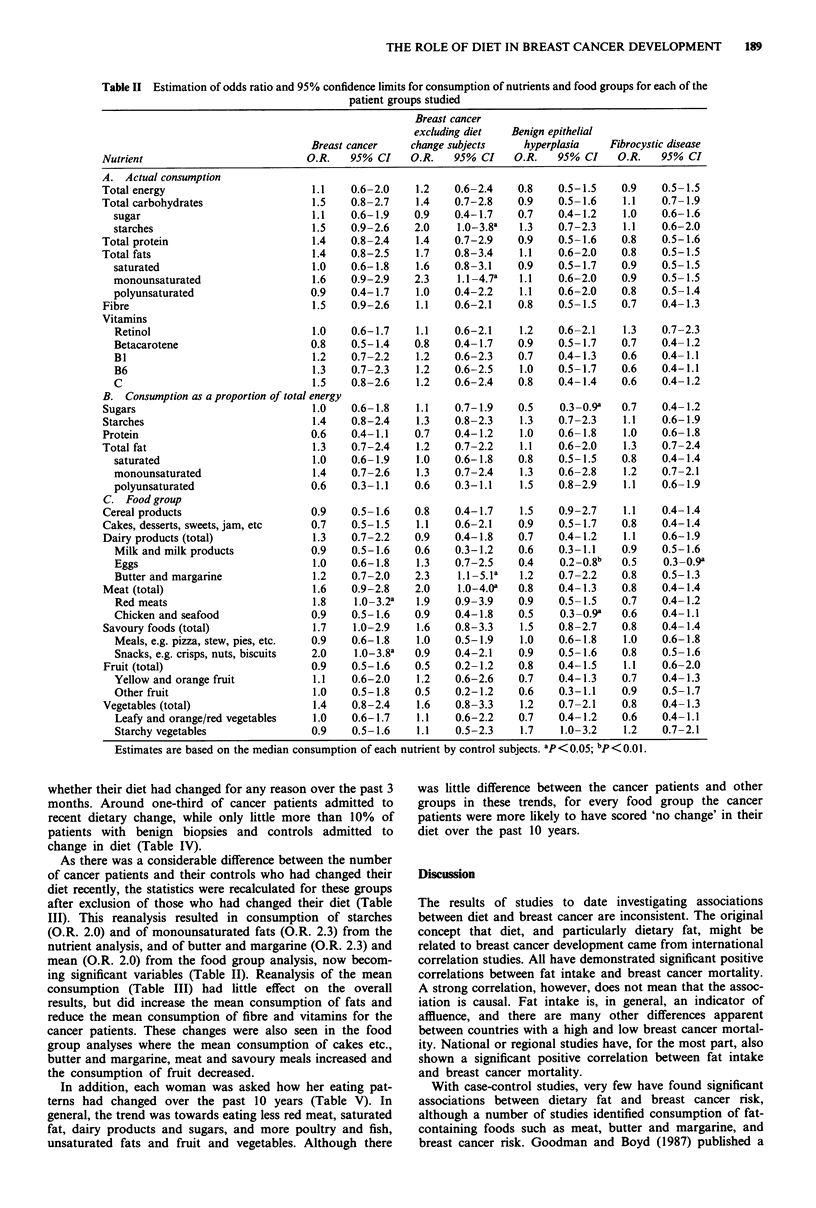

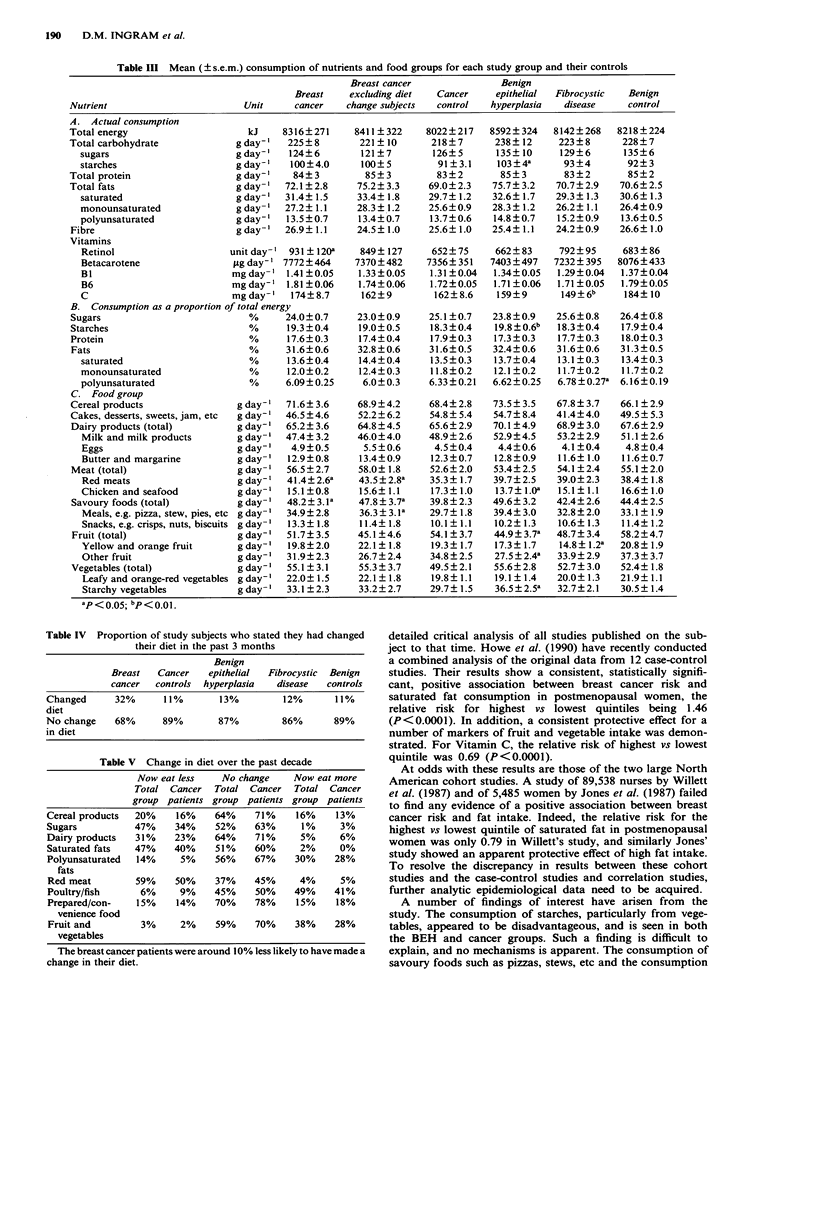

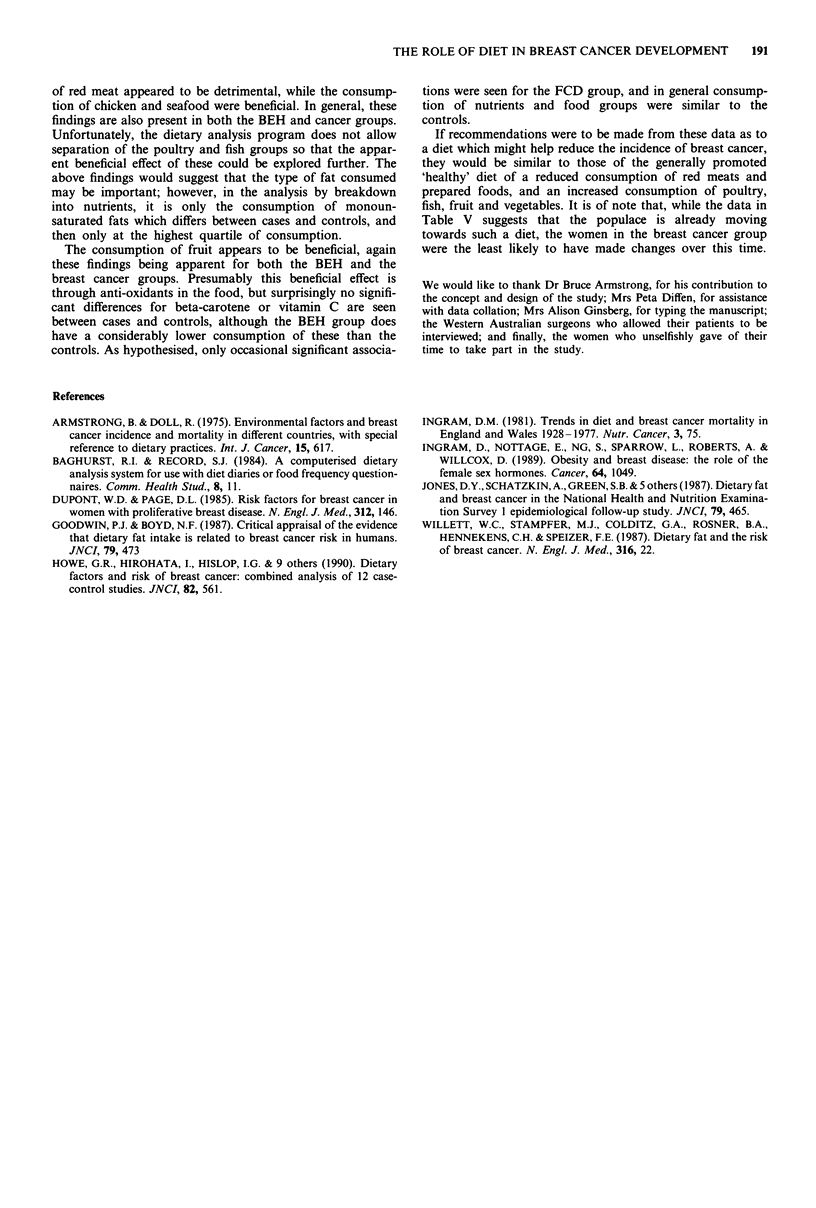


## References

[OCR_00913] Armstrong B., Doll R. (1975). Environmental factors and cancer incidence and mortality in different countries, with special reference to dietary practices.. Int J Cancer.

[OCR_00918] Baghurst K. I., Record S. J. (1984). A computerised dietary analysis system for use with diet diaries or food frequency questionnaires.. Community Health Stud.

[OCR_00923] Dupont W. D., Page D. L. (1985). Risk factors for breast cancer in women with proliferative breast disease.. N Engl J Med.

[OCR_00926] Goodwin P. J., Boyd N. F. (1987). Critical appraisal of the evidence that dietary fat intake is related to breast cancer risk in humans.. J Natl Cancer Inst.

[OCR_00931] Howe G. R., Hirohata T., Hislop T. G., Iscovich J. M., Yuan J. M., Katsouyanni K., Lubin F., Marubini E., Modan B., Rohan T. (1990). Dietary factors and risk of breast cancer: combined analysis of 12 case-control studies.. J Natl Cancer Inst.

[OCR_00936] Ingram D. M. (1981). Trends in diet and breast cancer mortality in England and Wales 1928-1977.. Nutr Cancer.

[OCR_00940] Ingram D., Nottage E., Ng S., Sparrow L., Roberts A., Willcox D. (1989). Obesity and breast disease. The role of the female sex hormones.. Cancer.

[OCR_00945] Jones D. Y., Schatzkin A., Green S. B., Block G., Brinton L. A., Ziegler R. G., Hoover R., Taylor P. R. (1987). Dietary fat and breast cancer in the National Health and Nutrition Examination Survey I Epidemiologic Follow-up Study.. J Natl Cancer Inst.

[OCR_00950] Willett W. C., Stampfer M. J., Colditz G. A., Rosner B. A., Hennekens C. H., Speizer F. E. (1987). Dietary fat and the risk of breast cancer.. N Engl J Med.

